# New Oligonucleotide Probes for ND-FISH Analysis to Identify Barley Chromosomes and to Investigate Polymorphisms of Wheat Chromosomes

**DOI:** 10.3390/genes7120118

**Published:** 2016-12-05

**Authors:** Shuyao Tang, Ling Qiu, Zhiqiang Xiao, Shulan Fu, Zongxiang Tang

**Affiliations:** 1 Province Key Laboratory of Plant Breeding and Genetics, Sichuan Agriculture University, Wenjiang, Chengdu 611130, China; tangshuyao705708@sina.com (S.T.); qiuling705@sina.com (L.Q.); xiaozq705@sina.com (Z.X.); 2 Institute of Ecological Agriculture, Sichuan Agricultural University, Wenjiang, Chengdu 611130, China

**Keywords:** barley, wheat, ND-FISH, chromosomal polymorphism, tandem repeats

## Abstract

Oligonucleotide probes that can be used for non-denaturing fluorescence in situ hybridization (ND-FISH) analysis are convenient tools for identifying chromosomes of wheat (*Triticum aestivum* L.) and its relatives. New oligonucleotide probes, Oligo-HvT01, Oligo-pTa71-1, Oligo-s120.1, Oligo-s120.2, Oligo-s120.3, Oligo-275.1, Oligo-275.2, Oligo-k566 and Oligo-713, were designed based on the repetitive sequences HVT01, pTa71, pTa-s120, pTa-275, pTa-k566 and pTa-713. All these probes can be used for ND-FISH analysis and some of them can be used to detect polymorphisms of wheat chromosomes. Probes Oligo-HvT01, Oligo-pTa71-1, Oligo-s120.3, Oligo-275.1, Oligo-k566 and Oligo-713 can, respectively, replace the roles of their original sequences to identify chromosomes of some barley (*Hordeum vulgare* ssp. *vulgare*) and the common wheat variety Chinese Spring. Oligo-s120.1, Oligo-s120.2 and Oligo-275.2 produced different hybridization patterns from the ones generated by their original sequences. In addition, Oligo-s120.1, Oligo-s120.2 and Oligo-s120.3, which were derived from pTa-s120, revealed different signal patterns. Likewise, Oligo-275.1 and Oligo-275.2, which were derived from pTa-275, also displayed different hybridization patterns. These results imply that differently arranged or altered structural statuses of tandem repeats might exist on different chromosome regions. These new oligonucleotide probes provide extra convenience for identifying some wheat and barley chromosomes, and they can display polymorphisms of wheat chromosomes.

## 1. Introduction

Fluorescence in situ hybridization (FISH) techniques have played an important role in modern molecular cytogenetics because they provide an useful tool for chromosome identification, which is an essential prerequisite for deducing the functions of chromosomes. Development of chromosome-specific probes is a key step for successful FISH analysis. Repetitive DNA sequences are often used as probes for FISH analysis on plant chromosomes because they can generate specific FISH signal patterns on individual chromosomes within a single species [1]. Tandem repeats including pAs1, pSc119.2, pTa71 and (AAG)_n_ are mainly used probes in FISH analysis of wheat (*Triticum aestivum* L.) and its relatives [2,3,4,5,6]. Additionally, some new tandemly repeated sequences, including pTa-s120, pTa-275, pTa-535, pTa-k566, pTa-713, etc., have been used as FISH probes to analyze wheat chromosomes [7]. These new repeated sequences are especially valuable probes for FISH analysis of wheat because they can produce distinctive bands on many chromosome arms [7]. However, conventional procedures of probe preparation using pAs1, pSc119.2, pTa71 as well as these newly developed probes, are time-consuming and labor-intensive. Recently, some oligonucleotide probes were developed based on the repetitive sequences pAs1, pSc119.2, pTa-535, pTa71, CCS1 and pAWRC.1 [8]. These oligonucleotide probes can replace the role of the repetitive sequences for FISH analysis of wheat and rye [8]. In addition, these oligonucleotides were shown to be suitable for non-denaturing fluorescence in situ hybridization (ND-FISH) analysis and the hybridization time was reduced to one hour [9,10]. Therefore, this technique provides a convenient way to use the oligonucleotide probes to analyze chromosomes of wheat and its relatives. In fact, ND-FISH has already been used to analyze plant telomeres [11]. Simple sequence repeats (SSR) were often used as probes for ND-FISH analysis of chromosomes of wheat and its relatives [12,13,14,15], as these SSR probes had been used to investigate chromosomes of *Drosophila melanogaster* and *Pinus sylvestris* [16,17]. However, SSR probes do not provide enough information for the confident identification of chromosomes of wheat and of its relatives. Consequently, in the present study, some new oligonucleotide probes were developed for ND-FISH analysis to identify wheat and barley (*Hordeum vulgare* ssp. *vulgare*) chromosomes.

## 2. Materials and Methods

### 2.1. Plant Materials

Three common wheat varieties, namely Chinese Spring (CS), Mianyang 11 (MY11), and Chuannong 27 (CN27) as well as three barley varieties (*Hordeum vulgare* ssp. *vulgare*), CNSimai 1, PI 328463 and PI 447312, were used to test the new oligonucleotide probes developed in this study. Wheat varieties CS, MY11, and CN27 were provided by our laboratory. The barley cultivar CNSimai 1 was kindly supplied by Professor Zongyun Feng, Agronomy College, Sichuan Agricultural University. Barley lines PI 328463 and PI 447312 were kindly supplied by Professor Zujun Yang, School of Life Science and Technology, University of Electronic Science and Technology of China.

### 2.2. Oligonucleotide Probe Development

Oligonucleotide probes were designed according to the repetitive sequences pTa71, HVT01, pTa-s120, pTa-275, pTa-k566 and pTa-713 [7,18,19]. The names and the sequences of these probes are listed in Table 1.

### 2.3. ND-FISH Analysis

Oligonucleotide probes were synthesized by Shanghai Invitrogen Biotechnology Co. Ltd. (Shanghai, China). The new developed oligonucleotides were 5′–end-labelled with 6-carboxyfluorescein (6-FAM) or 6-carboxytetramethylrhodamine (TAMRA) (Table 1). The chromosome spreads of materials were prepared through the methods described by Han et al. [20]. ND-FISH analysis was operated according to the methods described by Fu et al. [9]. In addition, (AAG)_6_, Oligo-pSc119.2-1 and Oligo-pTa535-1 [9] were also used to help identify wheat and barley chromosomes. Probe Oligo-pSc119.2-1 was 5′-end-labeled with TAMRA. Probes (AAG)_6_ and Oligo-pTa535-1 were 5′-end-labeled with Cyanine Dye 5 (Cy5). At least 10 metaphase cells were examined for each slide and only the chromosomes with strong or clear signals were selected.

### 2.4. Cloning of Original Repetitive DNA Sequences

To confirm that the oligonucleotide probes and their corresponding original repetitive sequences have a similar effect in identifying barley and wheat chromosomes, primer pairs were designed based on the sequences of HVT01, pTa-s120, pTa-275, pTa-k566 and pTa-713 (Table 2). The primer pairs listed in Table 2 were used to clone their corresponding repetitive DNA sequences by polymerase chain reaction (PCR). Genomic DNA (gDNA) from common wheat variety CS and the barley cultivar CNSimai 1 were extracted from leaf tissue according to the method described by Murray and Thompson [21]. For primer pairs 120, 275, 566 and 713, gDNA of CS was used as the template, and for primer pair HvT01, gDNA of barley cultivar CNSimai 1 was used as the template. The PCR reactions were carried out according to the methods described by Li et al. [22] with a slight modification in that the annealing temperature was 60 °C. The PCR products derived from each of the primer pairs were cloned and then sequenced according to the methods described by Tang et al. [23]. Sequence analysis was performed with the software DNAMAN (Ver. 4.0, Lynnon Corp., Quebec, QC, Canada) and Clustal X (ver. 2.0, Conway Institute UCD, Dublin, Ireland).

### 2.5. Denaturing FISH

The cloned repetitive sequences from CS and sequences of pTa71 were labeled with Texas Red-5-dUTP (Invitrogen, Carlsbad, CA, USA). The cloned repetitive sequences from barley were labeled with Alexa Fluor-488-5-dUTP (Invitrogen). Sequences of pTa71 were kindly provided by Professor Fangpu Han, Institute of Genetics and Developmental Biology, Chinese Academy of Science, Beijing, China. Denaturing FISH was carried out according to the procedure described by Tang et al. [8]. 

## 3. Results

### 3.1. New Oligonucleotide Probes for Identifying Barley Chromosomes

**Figure 1:**
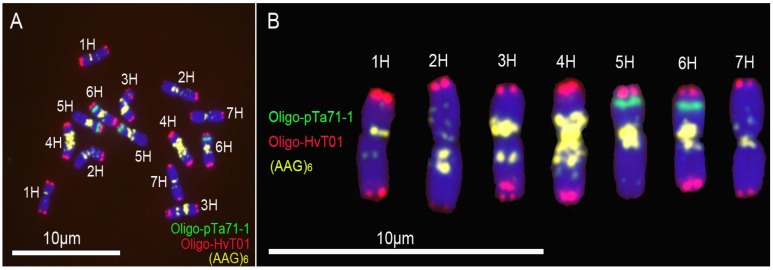
Non-denaturing in situ fluorescence hybridization (ND-FISH) analysis of root tip metaphase chromosomes of barley cultivar CNSimai 1: (**A**) Oligo-pTa71-1 (**green**), Oligo-HvT01 (**red**) and (AAG)_6_ (**yellow**) were used as probes; and (**B**) Karyotype of barley cultivar CNSimai 1. Chromosomes were counterstained with 4′,6-diamidino-2-phenylindole (DAPI) (**blue**). Scale bar: 10 μm.

The probes (AAG)_6_ and Oligo-HvT01 produced strong signals on all the seven pairs of barley chromosomes (Figure 1). Probe Oligo-pTa71-1 gave strong signals on the short arms of 5H and 6H chromosomes (Figure 1). The hybridization patterns of the probes (AAG)_6_, Oligo-HvT01 and Oligo-pTa71-1 on the chromosomes of cultivar CNSimai 1 are similar with the ones of (AAG)_7_, HVT01 and pTa71 on chromosomes of barley cultivar Igri [24].

### 3.2. New Oligonucleotide Probes for Identifying Wheat Chromosomes

Oligonucleotide probes Oligo-s120.1, Oligo-s120.2, Oligo-s120.3, Oligo-275.1, Oligo-275.2, Oligo-k566 and Oligo-713 were developed based on the original sequences of pTa-s120, pTa-275, pTa-k566 and pTa-713 [7] (Table 1).

**Figure 2:**
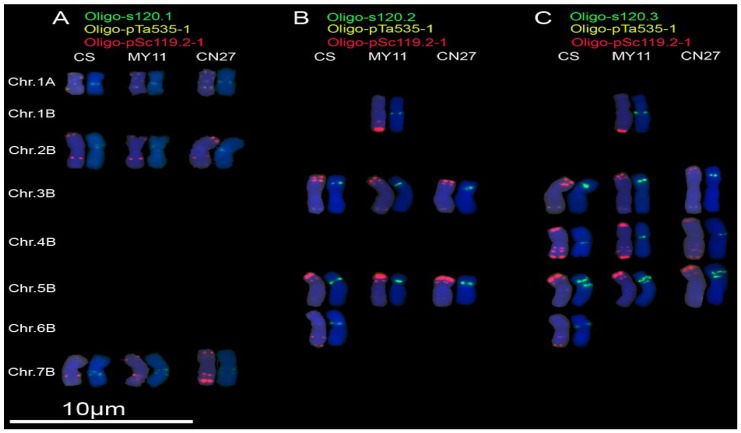
Oligo-s120.1 (**green**), Oligo-s120.2 (**green**) and Oligo-s120.3 (**green**) combined with Oligo-pTa535-1 (**yellow**) and Oligo-pSc119.2-1(**red**) were used as probes for ND-FISH analysis of root tip metaphase chromosomes of wheat varieties Chinese Spring (CS), Mianyang 11 (MY11), and Chuannong 27 (CN27): (**A**) Oligo-s120.1 hybridization patterns; (**B**) Oligo-s120.2 hybridization patterns; and (**C**) Oligo-s120.3 hybridization patterns. Only the chromosomes with strong or clear signals of Oligo-s120.1, Oligo-s120.2 or Oligo-s120.3 were cut and pasted. Chr.: chromosome. Chromosomes were counterstained with DAPI (**blue**).

Signals of Oligo-s120.1 could be observed on chromosomes 1A, 2B and 7B of CS, MY11 and CN27, (Figure 2A; Figure S1). Oligo-s120.2 produced signals on 3B, 5B and 6B chromosomes of CS, on 1B, 3B and 5B chromosomes of MY11, and on 3B and 5B chromosomes of CN27 (Figure 2B; Figure S2). The signals of Oligo-s120.3 could be observed on 3B, 4B, 5B and 6B chromosomes of CS, on 1B, 3B, 4B and 5B chromosomes of MY11, and on 3B, 4B and 5B chromosomes of CN27 (Figure 2C; Figure S3). Among the three probes Oligo-s120.1, Oligo-s120.2 and Oligo-s120.3, only the signal patterns of Oligo-s120.3 on chromosomes of CS are similar with the ones produced by pTa-s120 [7].

**Figure 3:**
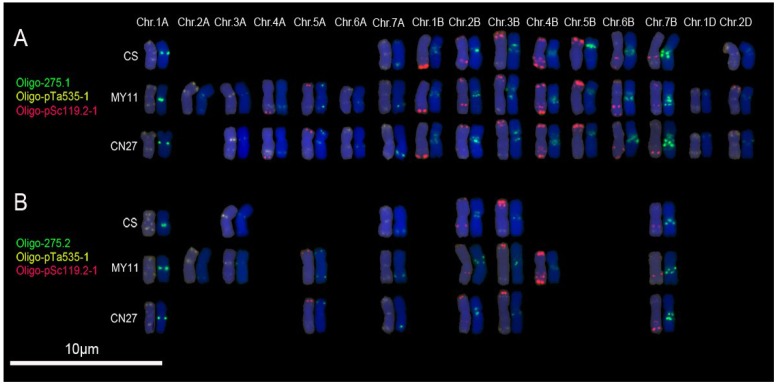
Oligo-275.1 (**green**) and Oligo-275.2 (**green**) combined with Oligo-pTa535-1 (**yellow**) and Oligo-pSc119.2-1 (**red**) were used as probes for ND-FISH analysis of root tip metaphase chromosomes of wheat varieties CS, MY11 and CN27: (**A**) Oligo-275.1 hybridization patterns; and (**B**) Oligo-275.2 hybridization patterns. Only the chromosomes with strong or clear signals of Oligo-275.1 or Oligo-275.2 were cut and pasted. Chr.: chromosome. Chromosomes were counterstained with DAPI (**blue**).

Oligo-275.1 produced hybridization signals on 1A, 7A, 2D and all the B-genome chromosomes of CS, on 1D, 2D and all the A- and B-genome chromosomes of MY11, and on 1A, 3A, 4A, 5A, 6A, 7A, 1D, 2D and all the B-genome chromosomes of CN27 (Figure 3A; Figure S4). It should be noted that signal patterns of Oligo-275.1 on the long arms of 5A chromosomes (5AL) of MY11 and CN27 are different. The signals of Oligo-275.1 on 5AL arms of MY11 are located at the sub-telomeric regions, however, on the 5AL arms of CN27, the signals are located at pericentromeric sites (Figure 3A; Figure S4). The hybridization signals of Oligo-275.2 could be observed on chromosomes 1A, 3A, 7A, 2B, 3B and 7B of CS, on chromosomes 1A, 2A, 3A, 5A, 7A, 2B, 3B, 4B and 7B of MY11, and on the chromosomes 1A, 5A, 7A, 2B, 3B and 7B of CN27 (Figure 3B; Figure S5). Again, it can be noted that the signal patterns of Oligo-275.2 on the 5A chromosomes between MY11 and CN27 are different (Figure 3B; Figure S5). No hybridization sites of Oligo-275.2 could be observed on 5AL arms of CN27, but the signals appeared on the 5AL arms of MY11 (Figure 3B; Figure S5). The hybridization patterns of Oligo-275.1 on chromosome of CS are similar with the ones of pTa-275 [7].

**Figure 4:**
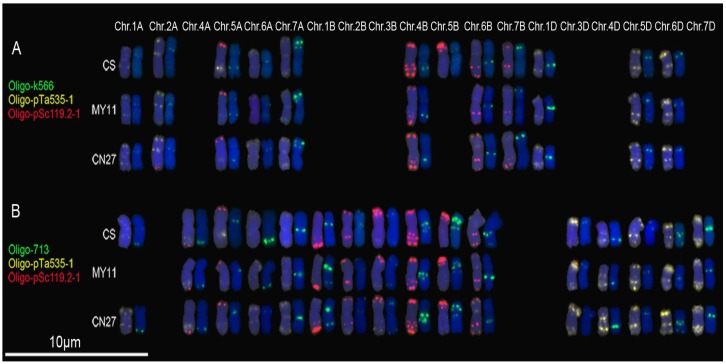
Oligo-k566 (**green**) and Oligo-713 (**green**) combined with Oligo-pTa535-1 (**yellow**) and Oligo-pSc119.2-1 (**red**) were used as probes for ND-FISH analysis of root tip metaphase chromosomes of wheat varieties CS, MY11 and CN27: (**A**) Oligo-k566 hybridization patterns; and (**B**) Oligo-713 hybridization patterns. Only the chromosomes with strong or clear signals of Oligo-k566 or Oligo-713 were cut and pasted. Chr.: chromosome. Chromosomes were counterstained with DAPI (**blue**).

Chromosomes 1A, 2A, 5A, 6A, 7A, 4B-7B, 1D, 5D and 6D of CS exhibited hybridization signals of Oligo-k566 (Figure 4A; Figure S6) and the fluorescence patterns are similar to those of pTa-k566 [7]. Oligo-k566 produced signals on chromosomes 1A, 2A, 5A, 6A, 7A, 4B, 6B, 7B, 1D, 5D and 6D of MY11 and CN27 (Figure 4A; Figure S6). The 5B chromosomes of both MY11 and CN27 contained no signals of Oligo-k566 (Figure 4A; Figure S6). Oligo-713 produced signals on 1A, 4A-7A, 1B-6B and 3D-7D chromosomes of CS and CN27 (Figure 4B; Figure S7). Signals of Oligo-713 could be observed on 4A-7A, 1B-6B and 3D-7D chromosomes of MY11, but chromosomes 1A of MY11 did not contain the signals (Figure 4B; Figure S7). It is worth noting that the signal patterns of Oligo-713 on 6A chromosomes of CS and MY11 are different from that of CN27, and on 1B and 6B chromosomes of MY11 and CN27 are different from the hybridization pattern of CS. Oligo-713 produced clear signals at the sub-telomeric regions of 1BL arms of MY11 and CN27, however, no signals were observed at these regions of CS (Figure 4B; Figure S7). In addition, there are two signal bands of Oligo-713 on 6BL of CS, and only one signal band of Oligo-713 could be observed on 6BL arms of MY11 and CN27 (Figure 4B; Figure S7). The signal patterns of Oligo-713 on the chromosomes of CS are similar with the ones of pTa-713 [7].

### 3.3. Cloning of Original Repetitive Sequences

Primer pairs HvT01 amplified a 263 base pair (bp) sequence that was named pMD-HvT01 (GenBank accesssion number KY014097). The sequence of pMD-HvT01 had 94.1% similarity to the original sequence of HVT01 (Figure S8A). Primer pairs 120 amplified a sequence, which was 392 bp long and was named pMD-120 (GenBank KY014098). The sequence of pMD-120 had 91.8% similarity to the original sequence of pTa-s120 (Figure S8B). Therefore, pMD-HvT01 and pMD-120 should respectively belong to their own repetitive sequence family. Primer 566R produced a sequence with 374 bp length and it was designated as pMD-566 (GenBank KY014099). This sequence had 80.3% similarity to the original sequence of pTa-k566 sequence (Figure S8C). The original pTa-k566 repetitive sequence contains two repeat units, which are about 350 bp long [7]. pMD-566 almost contained the 350 bp repeat unit, therefore, sequence pMD-566 also belongs to the pTa-k566 family. However, the sequences belong to the pTa-275 and pTa-713 families were unable to be successfully cloned. 

### 3.4. Hybridization Patterns of Oligonucleotide Probes and Their Corresponding Repetitive Sequence Families

Probes Oligo-pTa71-1 and pTa71 produced strong hybridization signals on the same chromosomal sites in each of the three barleys used in this study (Figure S9). The signal sites of probes pMD-HvT01 and Oligo-HvT01 on the chromosomes of the three kinds of barley were also same (Figure S10). These results indicate that the oligonucleotide probes Oligo-HvT01 and Oligo-pTa71-1 can respectively replace the roles of HVT01 and pTa71 to identify chromosomes of some barley varieties. Probes pMD-566 and Oligo-k566 had the same signal sites on chromosomes of wheat CS (Figure S11). The signals of pMD-120 and Oligo-s120.3 on chromosomes of wheat CS were similar, but little differences could be observed (Figure S11). No signals of pMD-120 were observed on chromosomes 4B, and Oligo-s120.3 produced weak signals (Figure S11). The signals of Oligo-s120.3 on pericentromeric regions of 5BL arms were very strong, however the signals of pMD-120 were weak (Figure S11). The hybridization patterns of Oligo-s120.3 on chromosomes of CS are more like the ones produced by pTa-s120 [7].

## 4. Discussion

### 4.1. Convenience of Oligonucleotide Probes in Analyzing Barley and Wheat Chromosomes

Repetitive DNA sequences including (AAG)_n_, HVT01 and pTa71 have been previously used as probes to analyze barley chromosomes by FISH methods [10,15,24,25,26]. These repetitive DNA sequences are useful in investigating the chromosomal structure of barley germplasm [10,15,24,25,26]. In earlier studies, the procedure of the preparation of probes HVT01 and pTa71 was time-consuming because it involved denaturing the DNA of the chromosome spreads and also that of the labeled probes. Our new non-denaturing FISH method has vastly simplified the hybridization technique but still retains the reliability and accuracy of chromosome identification. In the present study, Oligo-pTa71-1 and Oligo-HvT01 showed the same signal patterns as pTa71 and pMD-HvT01, respectively, on the chromosomes of some barley varieties (Figures S9 and S10). Therefore, oligonucleotide probes Oligo-HvT01 and Oligo-pTa71-1 appear to have the same roles as HVT01 and pTa71 in distinguishing the chromosomes of some barley varieties. Use of these oligonucleotides as probes for ND-FISH analysis of some barley chromosomes is much more convenient than the earlier procedure.

Repetitive DNA sequences pTa-s120, pTa-275, pTa-k566 and pTa-713 have been very useful in identifying wheat chromosomes in past study [7]. However, laborious DNA denaturing and hybridization procedures are needed to effectively utilize these repetitive DNA sequences [7]. We therefore attempted to develop oligonucleotides suitable for ND-FISH based on each of these repetitive sequences. In the present study, the sequences belonging to the pTa-275 and pTa-713 families were unable to be successfully cloned. A total of 60 sequences amplified by primer pairs 275, 275.1, 713 and 713.1 were randomly selected for sequencing, however, no suitable sequences were obtained. The sequence pMD-566 was selected from 30 sequences amplified by primer pairs 566 and 566.1. Therefore, it is clear that it is not easy to obtain some tandem repeats which can be used as probes for FISH analysis. Other oligonucleotide developed in this study provide a means of identifying wheat chromosomes more easily. Oligo-k566 and pMD-566 produced the same signal patterns on chromosomes of CS which means that Oligo-k566 (ND-FISH) can now replace the use of probe pMD-566 (denaturing FISH). The minute differences of signal patterns between Oligo-s120.3 and pMD-120 might be caused by the nucleotide changes in GA dinucleotide repeat region (Figure S8). In fact, the signal patterns of Oligo-s120.3 on chromosomes of CS are more like the ones produced by pTa-s120 [7]. Oligo-s120.3 can now be considered as a replacement for probe pTa-s120.

Although the repetitive DNA sequences belong to pTa-275 and pTa-713 families were unable to be cloned in this study, in contrast to the results obtained by Komuro et al. [7], the conclusion can now be drawn that Oligo-s120.3, Oligo-275.1, Oligo-k566 and Oligo-713 can replace the roles of their original sequences to identify the chromosomes of common wheat CS. Oligo-s120.1, Oligo-s120.2 and Oligo-275.2 can also help to identify wheat chromosomes. In addition, Oligo-275.1, Oligo-275.2 and Oligo-k566 are especially valuable for the unambiguous discrimination the orientation of wheat chromosomes 7A. Therefore, these new oligonucleotide probes provide extra convenience for identifying wheat chromosomes.

### 4.2. Oligonucleotide Probes Displaying Polymorphisms of Wheat Chromosomes

Many kinds of molecular markers have been used to investigate the genetic diversity of wheat. A few studies have detected the diversity of common wheat at the chromosome level by using FISH analysis [3,27]. In the present study, Oligo-s120.2 and Oligo-s120.3 displayed polymorphisms for 1B and 6B chromosomes among CS, MY11 and CN27 (Figure 2B,C). Oligo-275.1 displayed diversity of hybridization patterns for 2A, 3A, 4A, 5A, 6A and 1D chromosomes, and Oligo-275.2 displayed diversity of 2A, 3A, 5A and 4B chromosomes among the three common wheat cultivars (Figure 3). Polymorphisms of 5B chromosomes among CS, MY11 and CN27 were shown by Oligo-k566 (Figure 4A). Oligo-713 displayed polymorphisms of 1A, 6A, 1B and 6B chromosomes among the three common wheat cultivars (Figure 4B). Therefore, these new oligonucleotide probes can be used to conveniently investigate chromosomal diversification of different wheat cultivars and this might be useful for wheat breeding programs. C-banding technology has been used to investigate 460 polyploid wheat accessions and extensive chromosomal rearrangements including translocations and inversions were observed [28]. However, minor rearrangements of chromosomes may not be detected by C-banding technology [28]. FISH analysis can detect subtle structural variations of chromosomes [3,27]. Possibly, diversity of wheat cultivars reflected at the chromosomal level may lead to the association of FISH hybridization patterns with combinations of agriculturally useful genes. Oligonucleotide probes that can reflect polymorphisms across wheat chromosomes may be extremely helpful for future wheat breeding programs.

### 4.3. Oligonucleotide Probes Implying Different Structural Status of Tandem Repeats

Two oligonucleotide probes, Oligo-pTa535-1 and Oligo-pTa535-2, have been developed based on the tandem repeated sequence pTa-535, and probes Oligo-pTa535-1, Oligo-pTa535-2 and pTa-535 produced similar hybridization patterns on wheat chromosomes [7,8]. However, in the present study, some oligonucleotide probes produced different signal sites from the ones associated with their original sequences. The signal sites of both Oligo-s120.1 and Oligo-s120.2 were different from the ones of pTa-s120 [7]. The hybridization patterns produced by Oligo-275.2 and pTa-275 were also different [7]. The results obtained in this study and previous studies indicate that some tandem repeats might target regions with a slightly different structure on different chromosomal regions. In addition, Oligo-s120.1, Oligo-s120.2 and Oligo-s120.3 showed different hybridization patterns (Figure 2). Likewise, Oligo-275.1 and Oligo-275.2 also displayed different hybridization patterns (Figure 3). 

## 5. Conclusions

In conclusion, some new oligonucleotide probes have been developed in the present study and they can be used to conveniently identify chromosomes of some barley and wheat varieties using ND-FISH. Some of the new oligonucleotide probes revealed polymorphisms of wheat chromosomes. In addition, different signal sites produced by the oligonucleotide probes that were derived from the same original sequence imply that different distributions of the same tandem repeats might exist on different chromosome regions.

## Figures and Tables

**Table 1:** Name, sequence, applied amount and sources of oligonucleotide probes for Fluorescence in situ hybridization (FISH) analysis.

Probe Name	Sequence and Fluorochrome Label	The Amount of Each Slide (ng/Slide)	Sequences Used to Develop Probes (GenBank Accession Number)
Oligo-HvT01	Tamra-5′AAACT CGCAT TTTTG GCCTA TTCTG GCTAG TTCTG CATGC TATTG CTCAC TGATT TTGG3′	11.6	Barley relic DNA HVT01, tandemly repeated sequence (X16095.1)
Oligo-pTa71-1	6-FAM-5′GACGC GCGCC ATGGA AAACT3′	10.8	Wheat ribosomal DNA (rDNA) 25S-18S intergenic region EcoRI-BamHI fragment (X07841.1)
Oligo-s120.1	6-FAM-5′TATCG AGTGC GAGTG AGATA TGCAT GTGTA TGTGT GACCC AGGTG GATGG AGAGT TTGA3′	21.8	*Triticum aestivum* clone pTa-s120 FISH-positive repetitive sequence (KC290913.1)
Oligo-s120.2	6-FAM-5′GGAGA GGGAT GAACA AGGTT TTTGT GTCGG ATGCA TGCGA CAGAA TTGAA GATTG TCGG3′	21.4	
Oligo-s120.3	6-FAM-5′GAGAG AGAGA GAGAG AGAGA GAGAG AGAGA GAGAG AGAGA GAGAG AGAGA GA3′	19.0	
Oligo-275.1	6-FAM-5′TGCTA CTGCT GCTAC TGCTG CTACT CCTGC TACTG CTACT ACTGC TACTC CTGCT ACTC3′	24.6	*Triticum aestivum* clone pTa-275 FISH-positive repetitive sequence (KC290911.1)
Oligo-275.2	6-FAM-5′CTACT ACTAC TACTA CTACT ACTAC TACTA CTACT ACTAC TACTA CTACT ACTAC TACT3′	22.6	
Oligo-k566	6-FAM-5′ATCCT ACCGA GTGGA GAGCG ACCCT CCCAC TCGGG GGCTT AGCTG CAGTC CAGTA CTCG3′	23.3	*Triticum aestivum* clone pTa-k566 FISH-positive repetitive sequence (KC290904.1)
Oligo-713	6-FAM-5′GTCGC GGTAG CGACG ACGGA CGCCG AGACG AGCAC GTGAC ACCAT TCCCA CCCTG TCTA3′	23.0	*Triticum aestivum* clone pTa-713 FISH-positive repetitive sequence (KC290900.1)

**Table 2:** Primer pairs that were designed according to original repetitive sequences.

Primer Name *	Primer Sequence (5′-3′)	Sequences Used to Design Primer (GenBank Accession Number)
120F	AGGAGGGAAGAGCTCTGAGA	*Triticum aestivum* clone pTa-s120 FISH-positive repetitive sequence (KC290913.1)
120R	GCATAAAACACGACCTCCCC	
275F	TGCTACTACTGCTGCTCCT	*Triticum aestivum* clone pTa-275 FISH-positive repetitive sequence (KC290911.1)
275R	CAGTAGCAGTAGTAGCAGCAG	
275.1F	GCTACTCCTGCTACTCCTGC	
275.1R	CAGTAGCAGTAGTAGCAGCAG	
566F	TGCAATCCAGTACTCGCCTA	*Triticum aestivum* clone pTa-k566 FISH-positive repetitive sequence (KC290904.1)
566R	GTCGCTCTCCACTCAGTAGG	
566.1F	TCCAGTACTCGCCTAAGTTTGA	
566.1R	CTGGAATGCAGCTAAGCCTC	
713F	CCTCTGCCACCCTGTCTTAG	*Triticum aestivum* clone pTa-713 FISH-positive repetitive sequence (KC290900.1)
713R	TAGACAGGGTGGGAATGGTG	
713.1F	GCCACCCTGTCTTAGCGTA	
713.1R	GTAAGATAGACAGGGTGGGAATG	
HvT01F	CCTATTCTGGCTAGTTCTGCA	Barley relic DNA HVT01, tandemly repeated seq (X16095.1)
HvT01R	AGCATACAAAATTGGCTGGAGT	

* “F” denotes forward primer. “R” denotes reverse primer.

## Supplementary Materials

The following are available online at www.mdpi.com/2073-4425/7/12/118/s1, Figure S1: Oligo-s120.1 (green) combined with Oligo-pTa535-1 (yellow) and Oligo-pSc119.2-1(red) was used as probes for ND-FISH analysis. Figure S2: Oligo-s120.2 (green) combined with Oligo-pTa535-1 (yellow) and Oligo-pSc119.2-1(red) was used as probes for ND-FISH analysis. Figure S3: Oligo-s120.3 (green) combined with Oligo-pTa535-1 (yellow) and Oligo-pSc119.2-1(red) was used as probes for ND-FISH analysis. Figure S4: Oligo-275.1 (green) combined with Oligo-pTa535-1 (yellow) and Oligo-pSc119.2-1 (red) was used as probes for ND-FISH analysis. Figure S5: Oligo-275.2 (green) combined with Oligo-pTa535-1 (yellow) and Oligo-pSc119.2-1 (red) was used as probes for ND-FISH analysis. Figure S6: Oligo-k566 (green) combined with Oligo-pTa535-1 (yellow) and Oligo-pSc119.2-1 (red) was used as probes for ND-FISH analysis. Figure S7: Oligo-713 (green) combined with Oligo-pTa535-1 (yellow) and Oligo-pSc119.2-1 (red) was used as probes for ND-FISH analysis. Figure S8: Sequence alignment between cloned repetitive sequences and their corresponding original repetitive sequences. Figure S9: Oligo-pTa71-1 (green) and pTa71 (red) were used as probes for denaturing FISH analysis. Figure S10: Oligo-HvT01 (red) and pMD-HvT01 (green) were used as probes for denaturing FISH analysis. Figure S11: Oligo-s120.3 (green), Oligo-k566 (green), pMD-120 (red) and pMD-566 (red) were used as probes for denaturing FISH analysis.
