# Supplementary material for: New Oligonucleotide Probes for ND-FISH Analysis to Identify Barley Chromosomes and to Investigate Polymorphisms of Wheat Chromosomes
Source: Genes (Basel). 2016 Dec 5;7(12):118. doi: 10.3390/genes7120118 (PMC5192494; doi:10.3390/genes7120118)
Supplement: Supplementary file 1 [file genes-07-00118-s001.pdf]

# Supplementary Materials: New Oligonucleotide Probes for ND-FISH Analysis to Identify Barley Chromosomes and to Investigate Polymorphisms of Wheat Chromosomes

**Shuyao Tang, Ling Qiu, Zhiqiang Xiao, Shulan Fu and Zongxiang Tang**

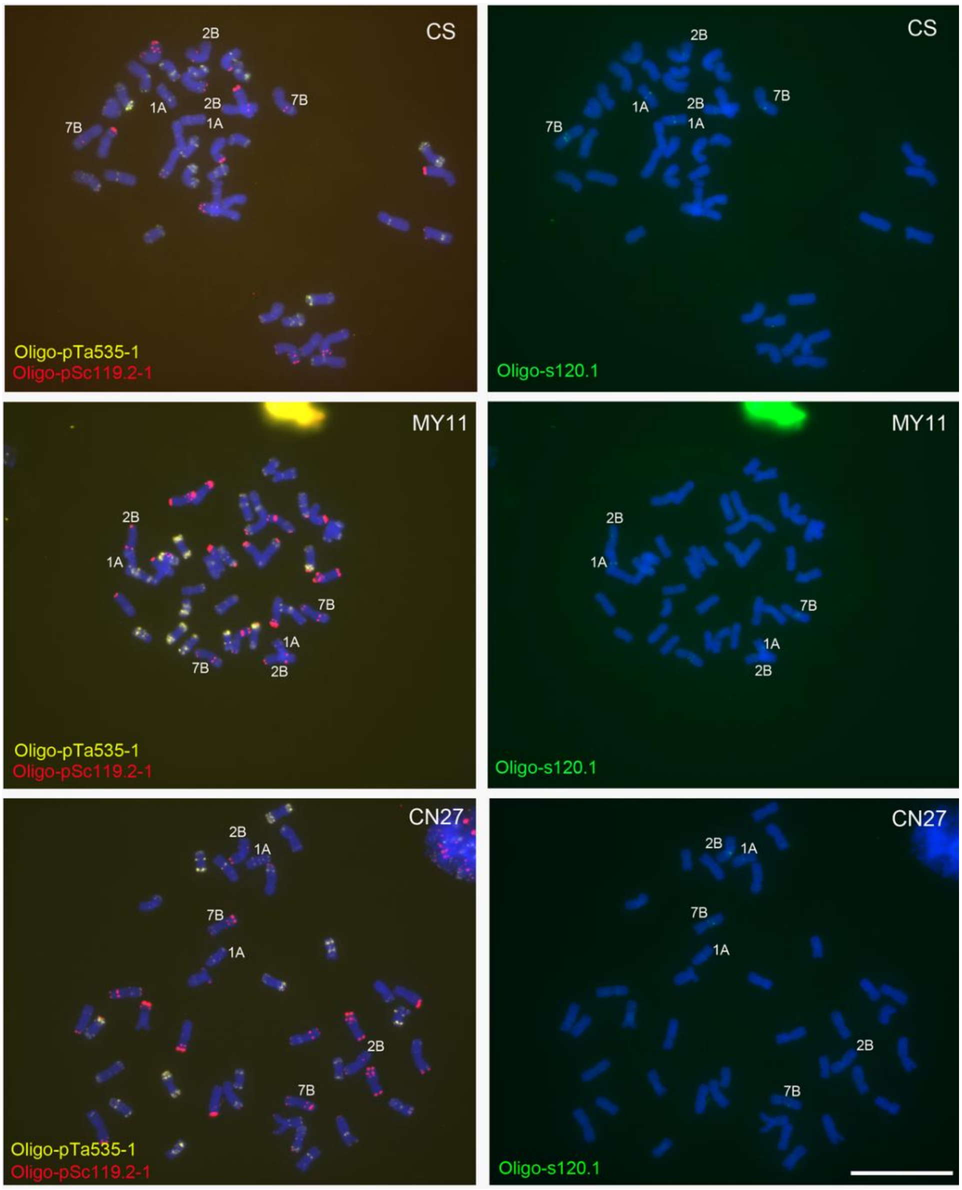


**Figure S1.** Oligo-s120.1 (**green**) combined with Oligo-pTa535-1 (**yellow**) and Oligo-pSc119.2-1(**red**) was used as probes for non-denaturing fluorescence in situ hybridization (ND-FISH) analysis of root tip metaphase chromosomes of wheat varieties Chinese Spring (CS), Mianyang 11 (MY11), and Chuannong 27 (CN27). Only the chromosomes with signals of Oligo-s120.1 were marked. Chromosomes were counterstained with with 4',6-diamidino-2-phenylindole (DAPI) (**blue**). Scale bar: 10 μm.

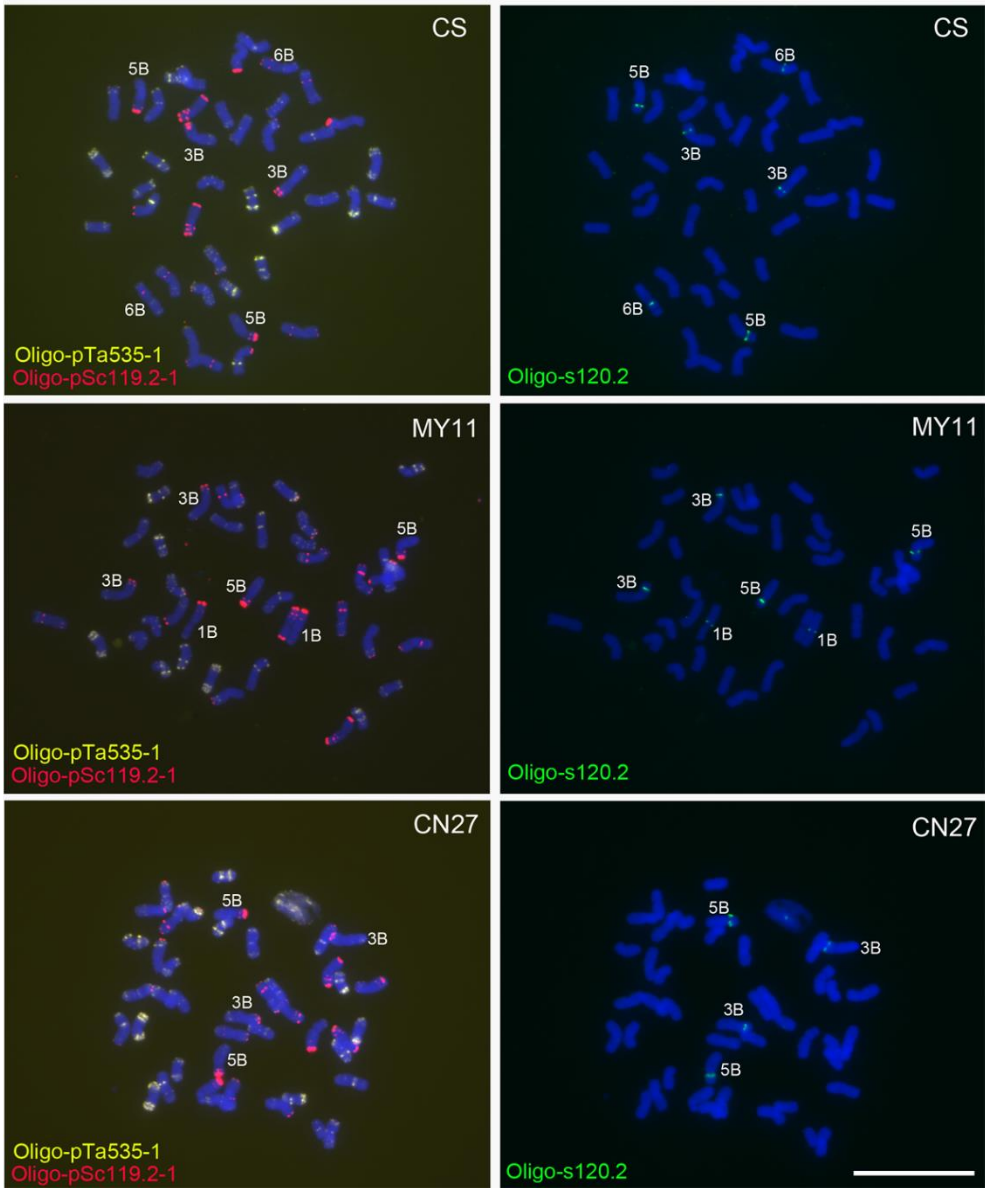


**Figure S2.** Oligo-s120.2 (**green**) combined with Oligo-pTa535-1 (**yellow**) and Oligo-pSc119.2-1(**red**) was used as probes for ND-FISH analysis of root tip metaphase chromosomes of wheat varieties CS, MY11 and CN27. Only the chromosomes with signals of Oligo-s120.2 were marked. Chromosomes were counterstained with DAPI (**blue**). Scale bar: 10 μm.

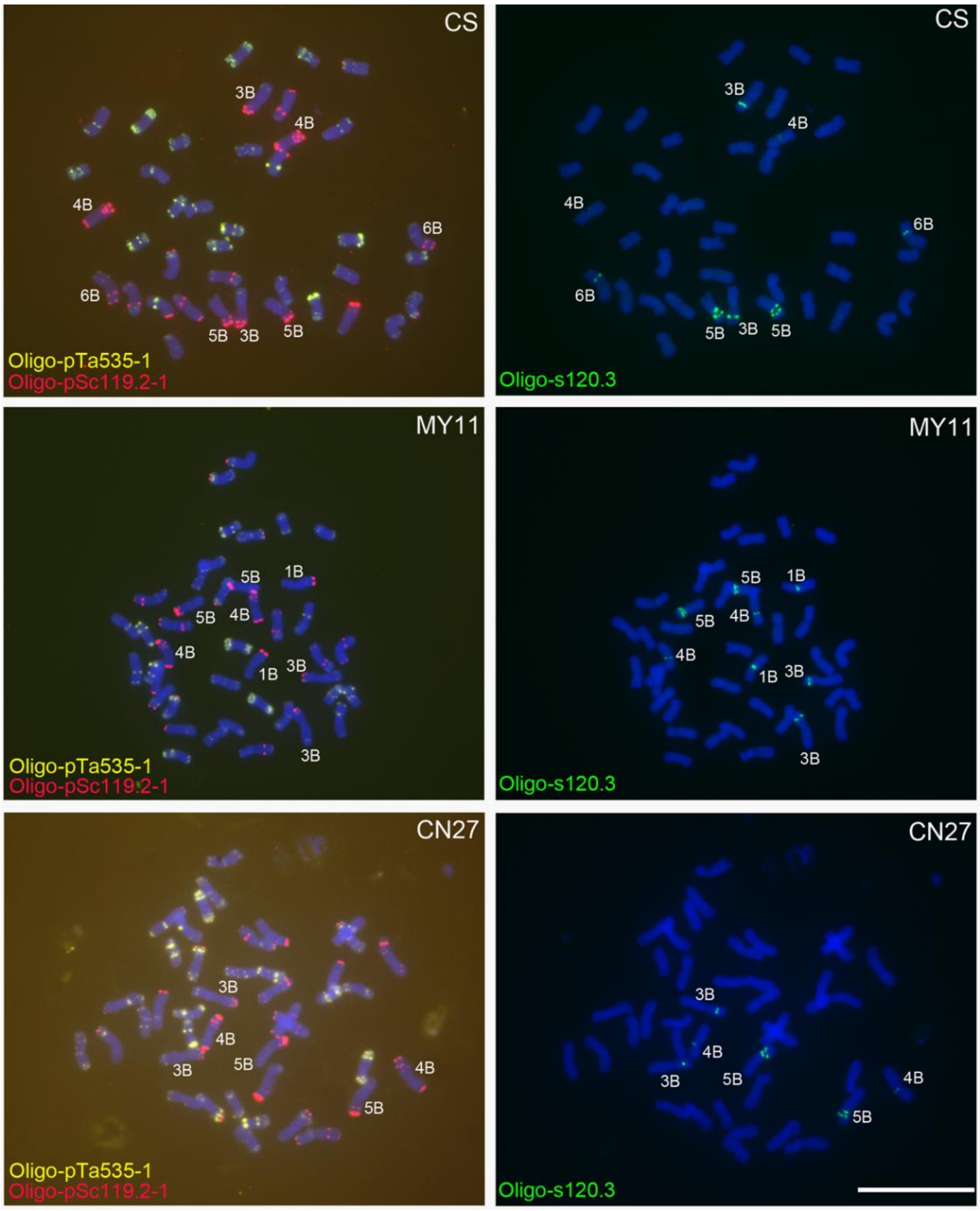


**Figure S3.** Oligo-s120.3 (**green**) combined with Oligo-pTa535-1 (**yellow**) and Oligo-pSc119.2-1(**red**) was used as probes for ND-FISH analysis of root tip metaphase chromosomes of wheat varieties CS, MY11 and CN27. Only the chromosomes with signals of Oligo-s120.3 were marked. Chromosomes were counterstained with DAPI (**blue**). Scale bar: 10 μm.

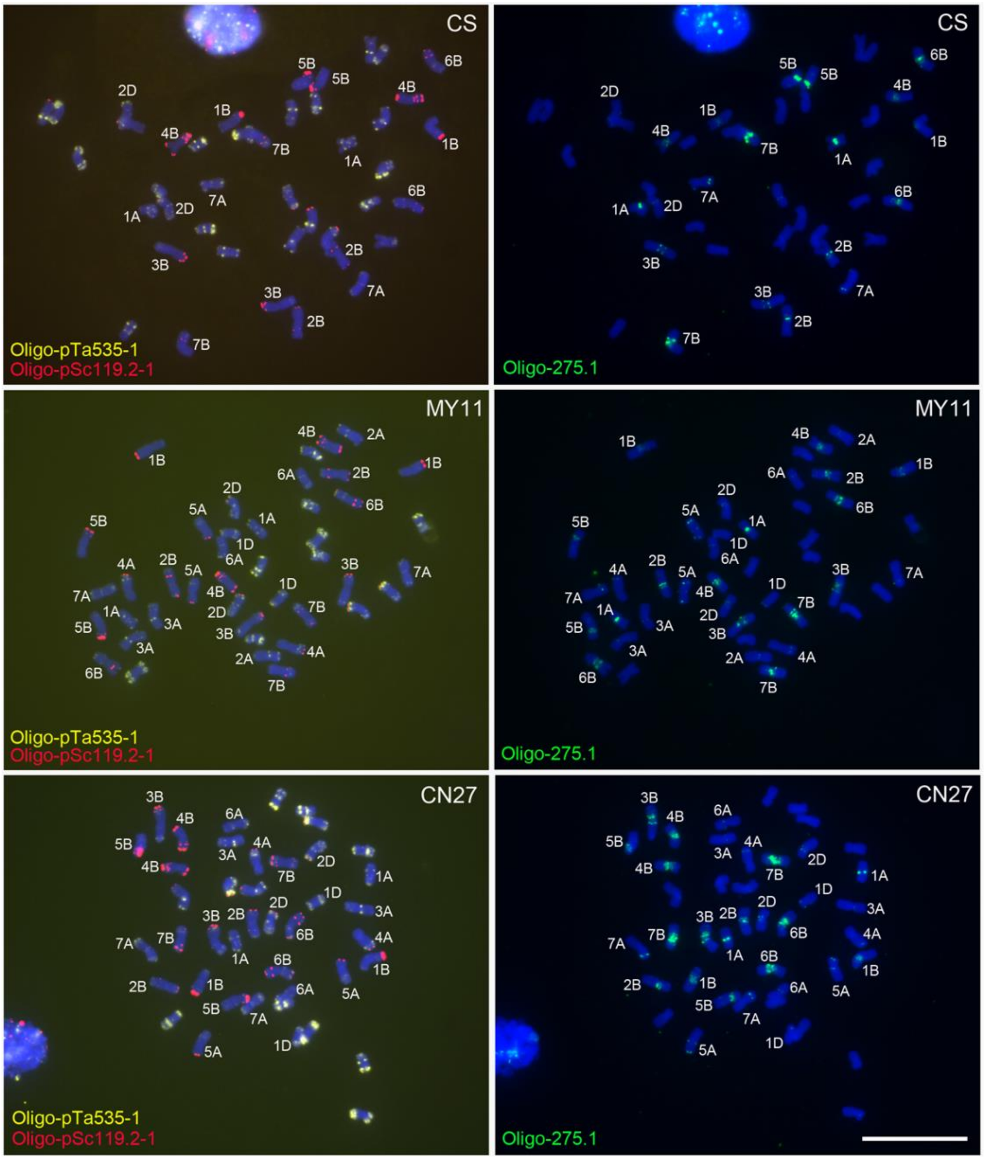


**Figure S4.** Oligo-275.1 (**green**) combined with Oligo-pTa535-1 (**yellow**) and Oligo-pSc119.2-1 (**red**) was used as probes for ND-FISH analysis of root tip metaphase chromosomes of wheat varieties CS, MY11 and CN27. Only the chromosomes with signals of Oligo-275.1 were marked. Chromosomes were counterstained with DAPI (**blue**). Scale bar: 10 μm.

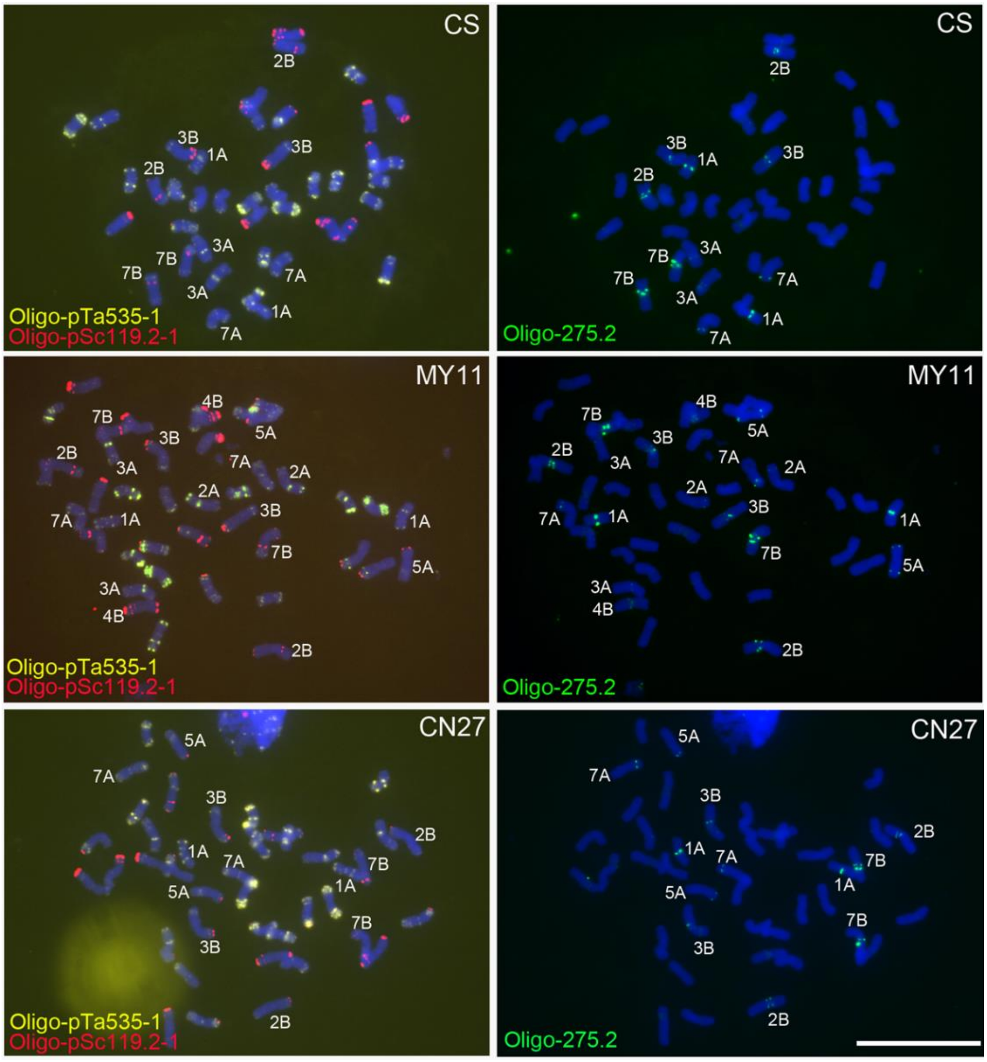


**Figure S5.** Oligo-275.2 (**green**) combined with Oligo-pTa535-1 (**yellow**) and Oligo-pSc119.2-1 (**red**) was used as probes for ND-FISH analysis of root tip metaphase chromosomes of wheat varieties CS, MY11 and CN27. Only the chromosomes with signals of Oligo-275.2 were marked. Chromosomes were counterstained with DAPI (**blue**). Scale bar: 10 μm.

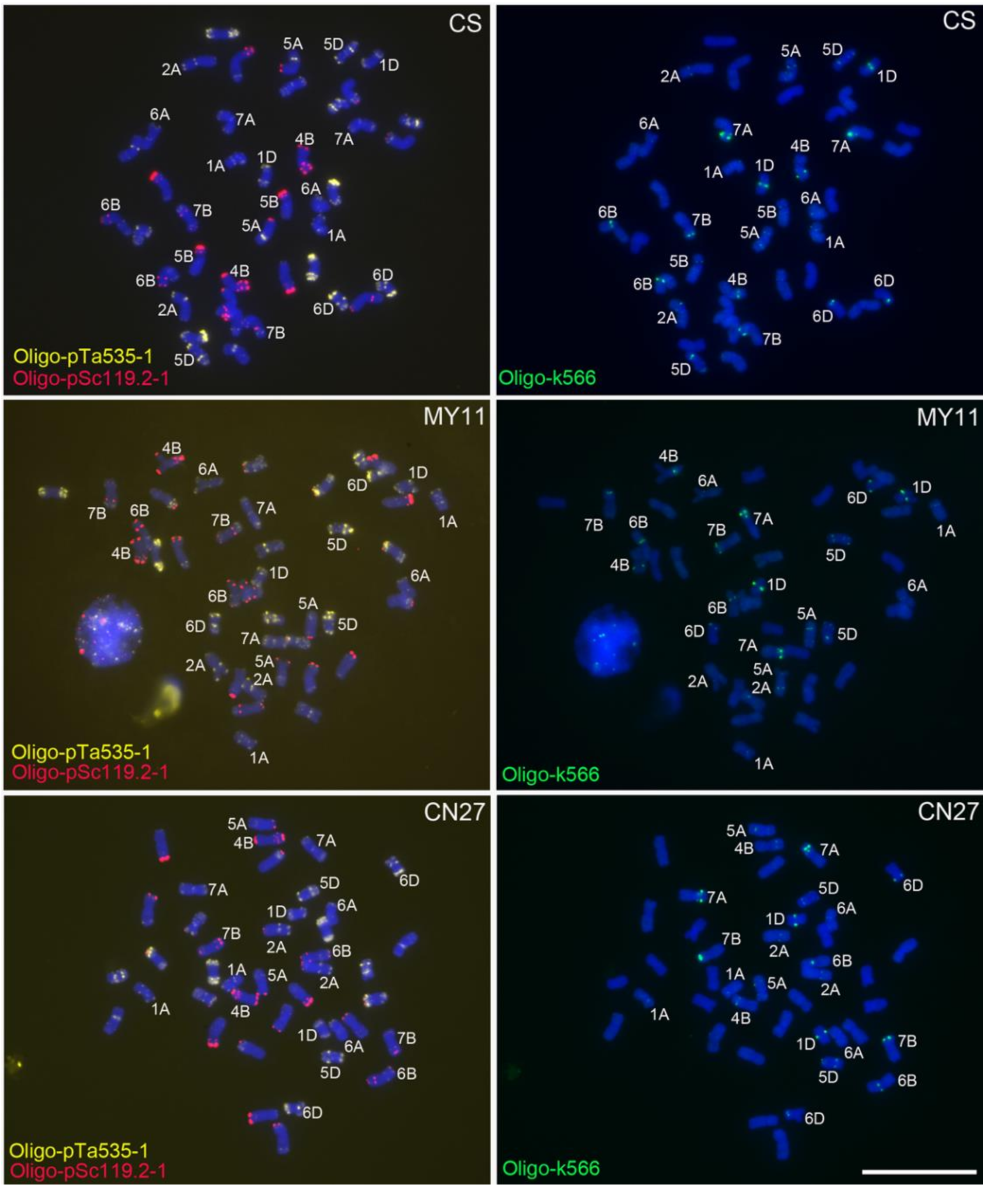


**Figure S6.** Oligo-k566 (**green**) combined with Oligo-pTa535-1 (**yellow**) and Oligo-pSc119.2-1 (**red**) was used as probes for ND-FISH analysis of root tip metaphase chromosomes of wheat varieties CS, MY11 and CN27. Only the chromosomes with signals of Oligo-k566 were marked. Chromosomes were counterstained with DAPI (**blue**). Scale bar: 10 μm.

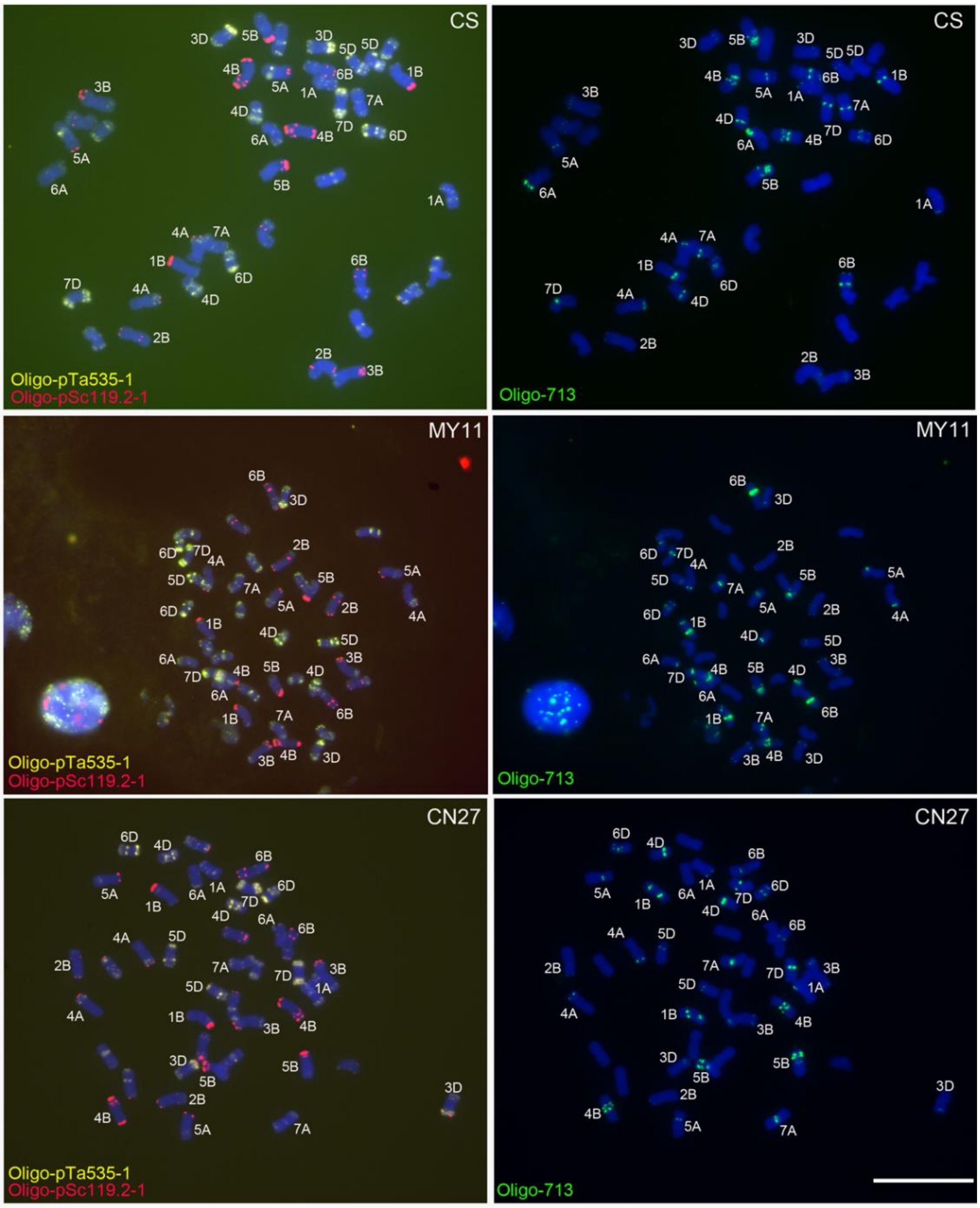


**Figure S7.** Oligo-713 (**green**) combined with Oligo-pTa535-1 (**yellow**) and Oligo-pSc119.2-1 (**red**) was used as probes for ND-FISH analysis of root tip metaphase chromosomes of wheat varieties CS, MY11 and CN27. Only the chromosomes with signals of Oligo-713 were marked. Chromosomes were counterstained with DAPI (**blue**). Scale bar: 10 μm.

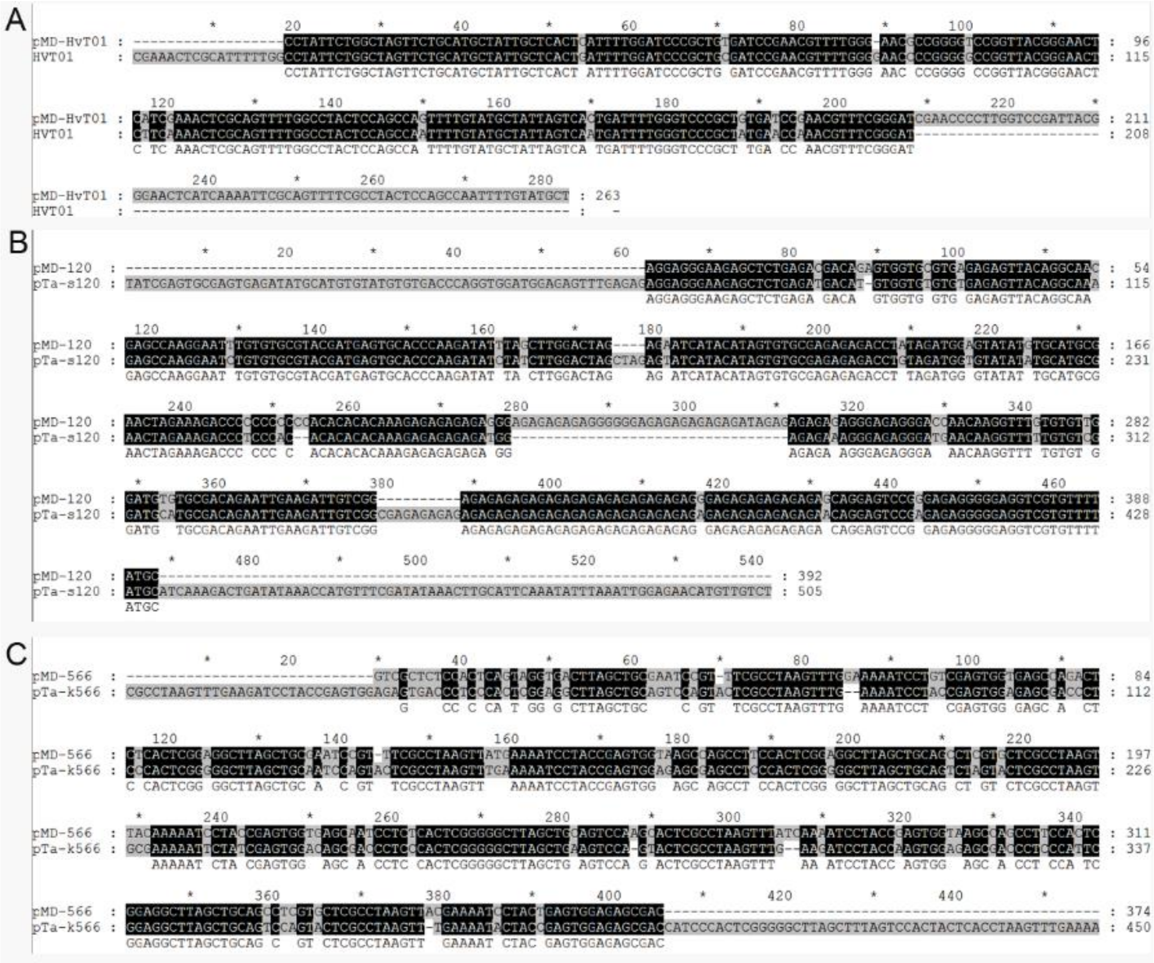


**Figure S8.** Sequence alignment between cloned repetitive sequences and their corresponding original repetitive sequences: (**A**) alignment between pMD-HvT01 and HVT01; (**B**) alignment between pMD-120 and pTa-s120; and (**C**) alignment between pMD-566 and pTa-k566.

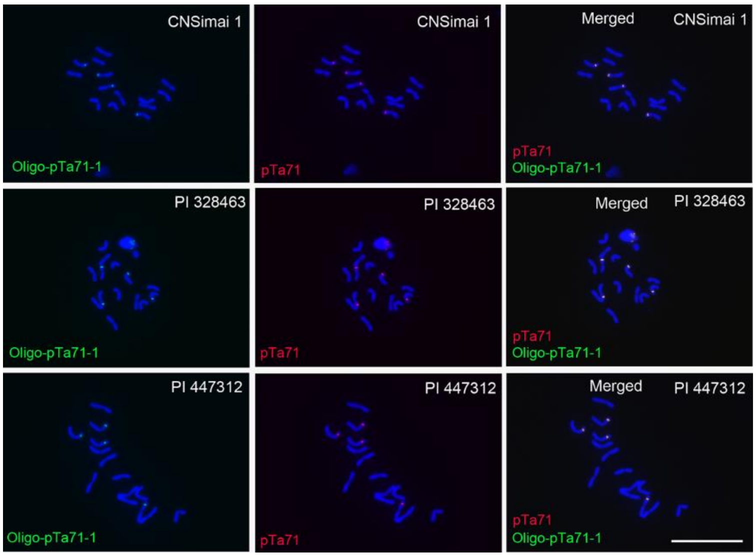


**Figure S9.** Oligo-pTa71-1 (**green**) and pTa71 (**red**) were used as probes for denaturing FISH analysis of root tip metaphase chromosomes of three barley varieties. Chromosomes were counterstained with DAPI (**blue**). Scale bar: 10 μm.

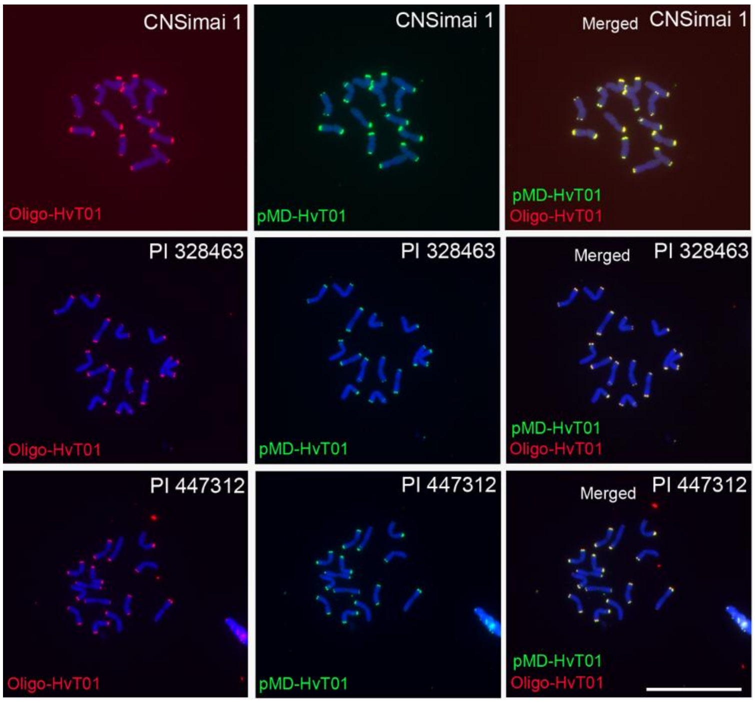


**Figure S10.** Oligo-HvT01 (**red**) and pMD-HvT01 (**green**) were used as probes for denaturing FISH analysis of root tip metaphase chromosomes of three barley varieties. Chromosomes were counterstained with DAPI (**blue**). Scale bar: 10 µm.

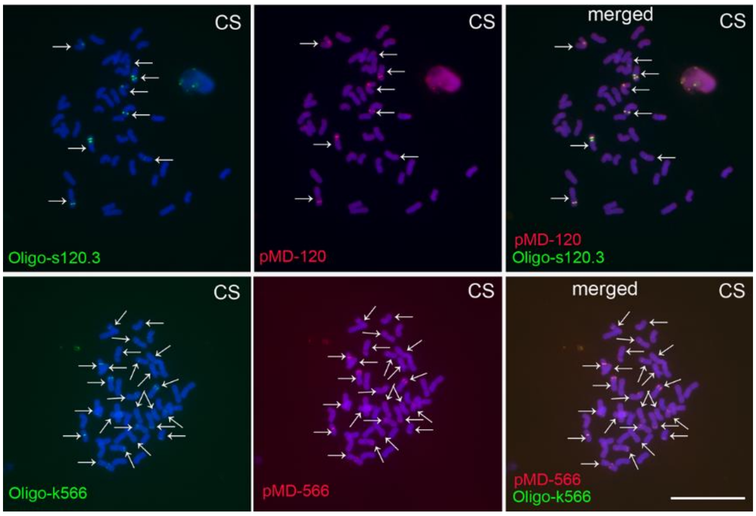


**Figure S11.** Oligo-s120.3 (**green**), Oligo-k566 (**green**), pMD-120 (**red**) and pMD-566 (**red**) were used as probes for denaturing FISH analysis of root tip metaphase chromosomes of wheat CS. Arrows indicate the chromosomes with hybridization signals. Chromosomes were counterstained with DAPI (**blue**). Scale bar: 10 µm.
